# Medical Society of the County of Erie

**Published:** 1898-03

**Authors:** F. C. Gram


### Society Proceedings.

### MEDICAL SOCIETY OF THE COUNTY OF ERIE.

*Special Meeting, held February 13, 1898.*

REPORTED BY F. C. GRAM, M. D., Secretary

THE Medical Society of the County of Erie met in special session Sunday, February 13, 1898, in the rooms of the Buffalo Academy of Medicine.

The president, Dr. LUCIEN HOWE, called it to order at 4.15 P. M. In announcing the death of Dr. John Cronyn, which had occurred February 11, 1898, he said that it was especially proper in this instance to do honor to the memory of a man who was deservedly prominent among his fellows. His natural ambition and his studious determination to excel brought proper honors. The institution of the Niagara University, the child of his brain, will stand as a monument to him or as a lengthened shadow of the man; another one will be the monument engraved upon the minds of his patients. "Death laid his hand gently upon his harp to deaden its vibrations."

On motion the president appointed as a committee on memorial Drs. Herman Mynter, Thomas Lothrop and H. R. Hopkins.

Dr. A. A. HUBBELL, in expressing his personal tribute, said that for the past fifteen years he had been intimately associated with the deceased, which had resulted to his interest and pleasure. Dr. Cronyn began his practice long before the days of anesthesia. His early contemporaries were such men as Flint, Hamilton, White and others, among whom he easily held his own. He constantly strove to excel. He kept in close touch with all progress. Always attentive at the meetings of our local societies, he was also related to state and national organisations. He was the central figure of the medical department of Niagara University, which he instituted not from selfish motives, but because he felt it was time to place medical education on a higher standard. His many good qualities stand out for us to emulate.

Dr. MYNTER, of the committee, then presented the following memorial:

IN MEMORIAM JOHN CRONYN, M. D.—1826-1898.

The Medical Society of the County of Erie has heard with profound sorrow of the death of one of its oldest and most valued members, Dr.

✓ pt. p. 62

John Cronyn, and it desires to place on its records its appreciation of his high professional attainments, his great services for higher medical education, his pure, modest, unassuming and christian life—a life that well may be taken as an example by the rest of the profession.

Dr. Cronyn was born in Ireland in 1826, but received his education at Toronto, where he was graduated in medicine. He practised in Fort Erie for nine years and removed to Buffalo in 1859, where he has since lived and labored. He quickly made a name for himself by his indefatigable work, his tireless industry, and close study of scientific medicine—a name he maintained by the same means—till he died in the harness.

He has held a number of appointments with credit to himself and honor to the bodies which greeted him. For eight years he acted as marine surgeon to Buffalo. For thirty-five years he has been first surgeon and then chief physician to the Sisters of Charity hospital and has seen it grow, largely under his fostering care and by his wise counsel, to the great and magnificent institution it now is. He has been president of the New York State Medical Association, of the Medical Society of the County of Erie and of the Surgical Association, the predecessor of the Buffalo Academy of Medicine, of the medical department of Niagara University, and occupied, from its foundation, the chair of principles and practice of medicine and was president of the faculty. He was an honorary member of Ontario Medical Association and received the degrees of Ph. D. and LL.D. from Niagara University for distinguished services in the cause of higher medical education ; a member and at one time president, of the board of managers of the Buffalo state hospital.

These are a few of the well-deserved honors that came to him and which he valued simply as an expression of public confidence. As a man he was a shining example of a courteous gentleman of the old school, but too rarely found in our busy days. He was a faithful friend and a wise counselor to the many young physicians educated under his eyes and under his guiding mind.

Quick to resent an injustice or insult, he was still quicker to forgive and forget. His mind was stored with facts and information from his incessant reading in all the branches of scientific medicine, he was a regular attendant at state, county and city medical societies, always ready in his courteous and gentle way to discuss any subject and always spreading new light in debate. As a lecturer, particularly in clinical medicine, he was painstaking, clear and scientific, never allowing private affairs to interfere with his official duties, always at hand at the appointed hour.

Dr. Cronyn believed most thoroughly in the efficacy of drugs and he gave his students his reasons for employing them in a way that was convincing and interesting. Who shall mention his relations to his patients (and among them a great many of the poor), but they themselves? His courteous, dignified, gentle appearance in the sick-room was a remedy in itself. He was a magnificent example of the old family physician and friend, maintaining always his patients' love and affection.

He was benevolent, with a kind heart and a generous hand to the sick, the sorrowful and the poor and he did not let his right hand know what his left gave away. As a citizen he was interested in the public welfare, an upright, virtuous, model man; as a friend he was faithful, considerate and loyal.

Surely the world is richer for such a life and well may we point with pride to the fact that he lived and labored among us for almost half a century and hope that we, the younger members of the profession, may emulate him in his virtues, his studiousness and his public spirit.

The society extends its heartfelt sympathy to his bereaved family, with the scriptural quotation: "Well done, good and faithful servant! enter thou into the joy of thy Lord."

HERMAN MYNTER,
THOMAS LOTHROP,
HENRY R. HOPKINS,
*Committee.*

Dr. Hopkins, in moving its adoption, took occasion to refer to his long association with the deceased. He began his practice in the immediate neighborhood of Dr. Cronyn, since which time their lines had lain along similar courses. In consequence of this the speaker could vouch for every statement contained in the memorial. He had borne himself even better than the memorial testifies. He was one of the old family physicians. He was an expert surgeon in his day, and yet he was known among the present generation only as a general practitioner. There was in his life also a sense of loftiness and he had a profound love for music, art and religion.

Dr. F. W. Abbott said that his acquaintance with the deceased began in 1865, while a resident physician in the Sisters of Charity hospital. He was always impressed with Dr. Cronyn's uniform courtesy and his aptitude to impart instruction. In the departure of so many of our members one thought presented itself strongly to the speaker: "Be ye also ready."

Dr. Conrad Diehl, the mayor, wished that he were better able to express his feelings at the loss of a fellow practitioner whom he had known since 1862, and with whom he had since been more or less associated. He was a true physician—charitable, honest and religious.

Dr. J. B. COAKLEY said that Dr. Cronyn's life has been fraught with noble usefulness. Respect for the rights and tenderness for the feelings of others stamped his conduct everywhere. He came up fully to Sidney's definition of a gentleman: "High thoughts seated in a heart of courtesy." Dr. Cronyn has been deservedly honored, loved and trusted by the people of this city. The speaker knew of no better legacy that a father could leave his household, or a patriot leave his country, than such a record as Dr. Cronyn left to attest his virtues.

Dr. W. C. CALLANAN had known the deceased since his own boyhood and spoke of his excellent qualities.

Dr. INGRAHAM said he had been intimately associated with Dr. Cronyn for fifteen years and the more he knew him the better he loved him. He was one of the most tolerant and least bigoted of physicians.

Dr. GROSVENOR mentioned the exceeding studiousness of Dr. Cronyn and his marvelous knowledge of medical literature.

The memorial was then adopted.

The following telegram was received by the president:

TONAWANDA, N. Y., February 13, 1898.

*Dr. Lucien Howe, President of the Medical Society of the County of Erie, 183 Delaware Avenue:*

I cannot express my sorrow at the sad loss of Dr. Cronyn. Our kind, hearty friend and physician is gone. We have no one to take his place. FREDERICK F. HOYER.

On motion the members decided to meet at 9.30 Monday morning, February 14th, in the grand court of Ellicott Square, and attend the funeral in a body.

The meeting then adjourned.

